# Supplementary figures and images for: Integrated analysis of lncRNAs, mRNAs, and TFs to identify network modules underlying diterpenoid biosynthesis in Salvia miltiorrhiza
Source: PeerJ. 2023 May 9;11:e15332. doi: 10.7717/peerj.15332 (PMC10178227; doi:10.7717/peerj.15332)

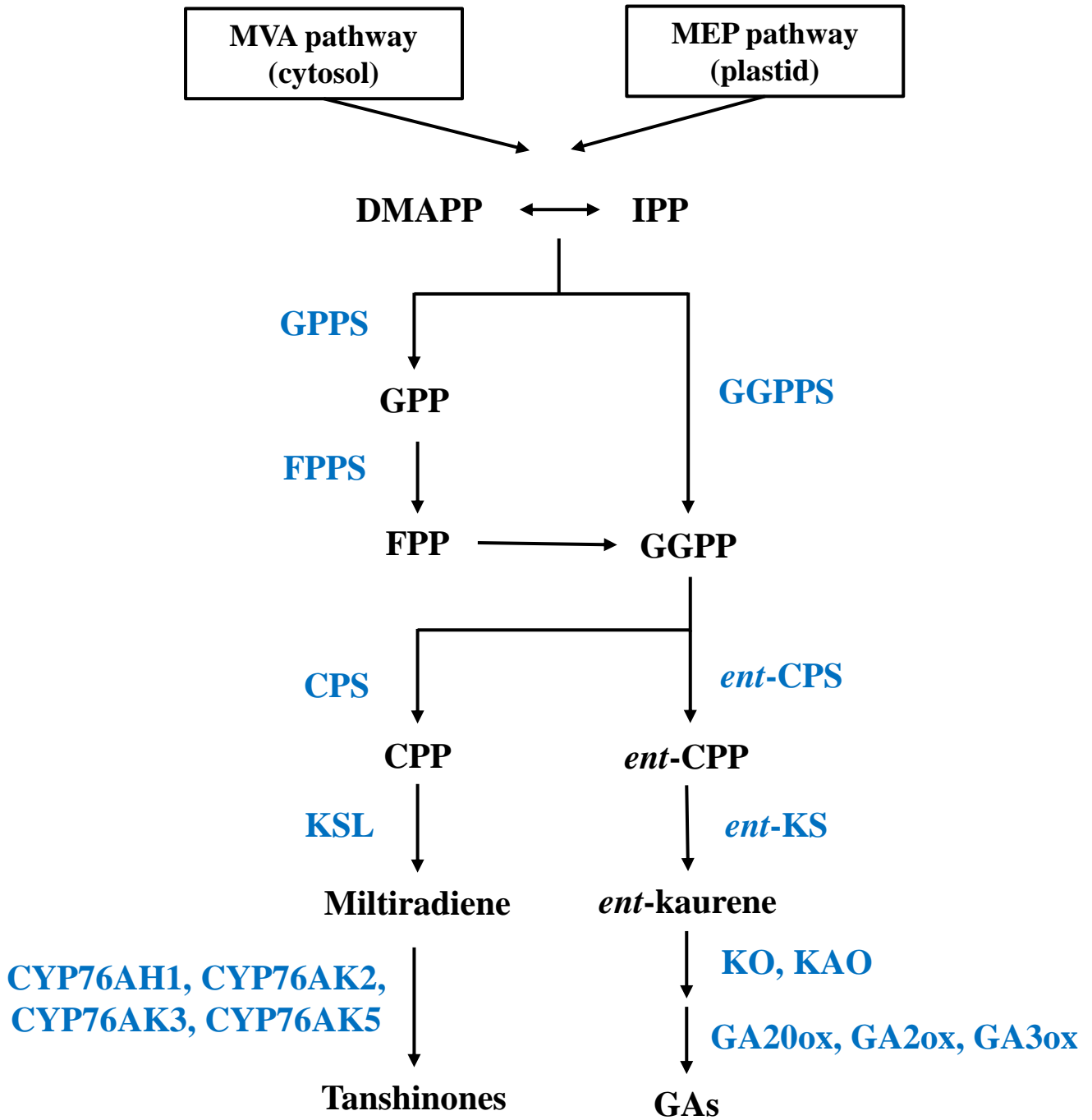

Supplement: Supplemental Information 1 — GGPPS, geranylgeranyl diphosphate synthase; GPPS, geranyl diphosphate synthase; FPPS, farnesyl diphosphate synthase; CPS, copalyl diphosphate synthase; ent-CPS, ent-copalyl diphosphate synthase; KS, kaurene synthase; ent-KS, ent-kaurene synthase; CYP76AH1; CYP76AK2; CYP76AK3; CYP76AK5; KO , ent-kaurene oxidase; KAO, ent-kaurenoic acid oxidase; GA2ox, GA 2-oxidase; GA3ox, GA 3-oxidase; GA20ox, GA 20-oxidase. [file peerj-11-15332-s001.pdf]

(A)

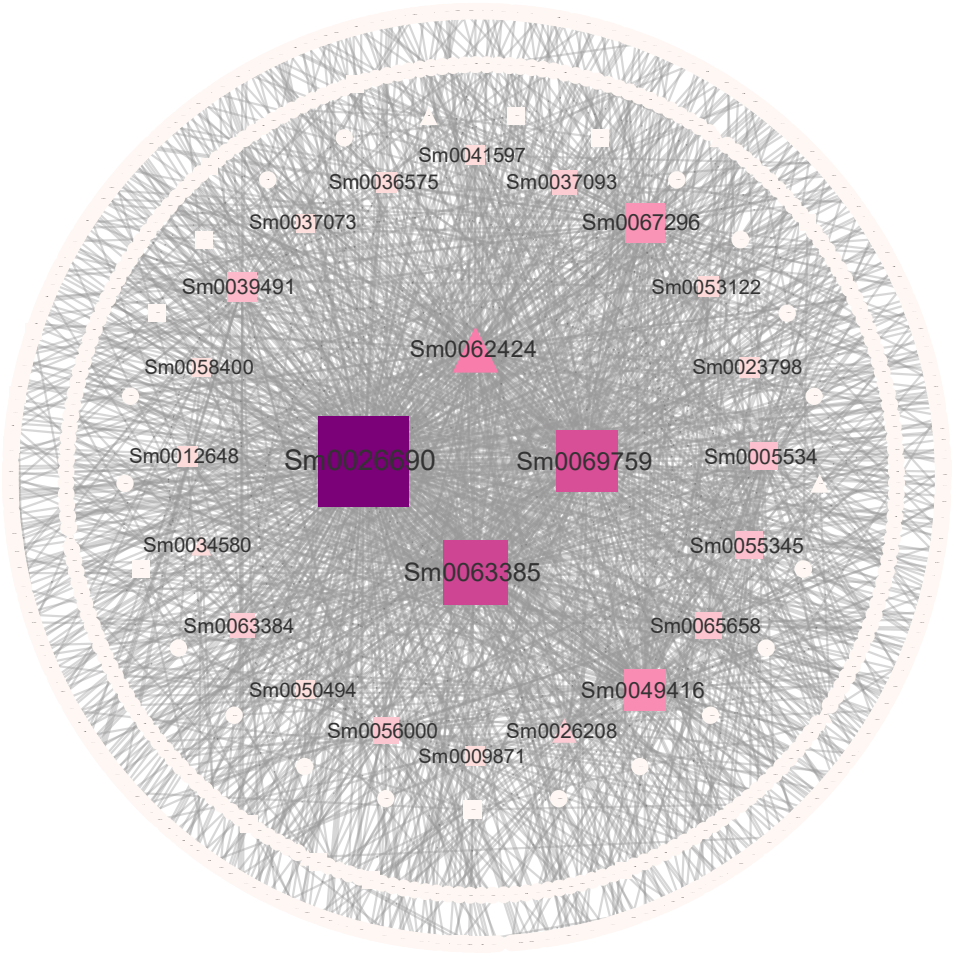

(B)

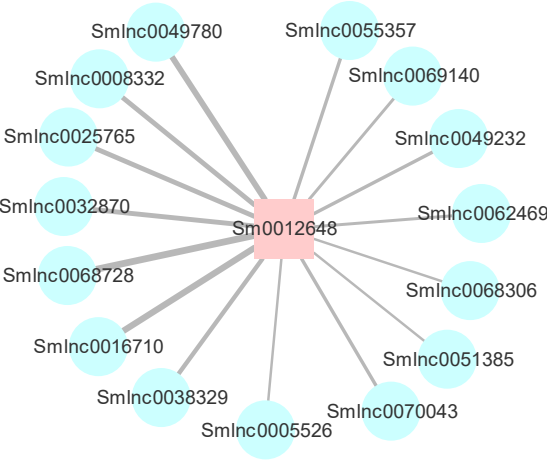

(C)

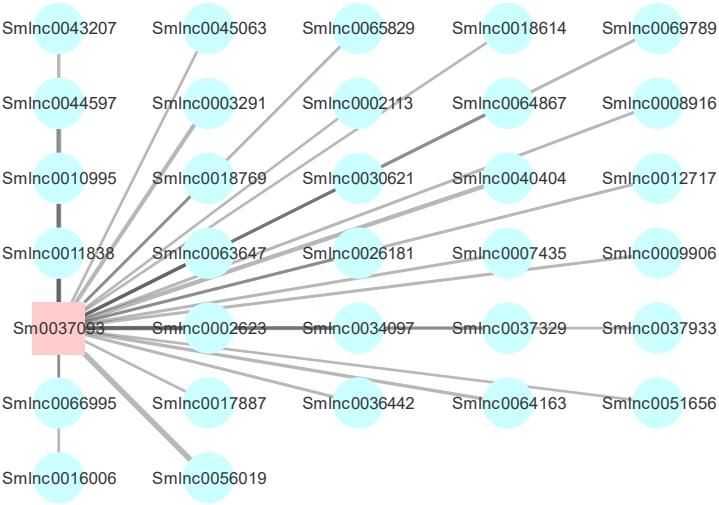

Supplement: Supplemental Information 2 — (A) Co-expression network with —PCC—≥ 0.8. (B) Co-expression network of the Sm0012648 hub genes. (C) Co-expression network of the Sm0037093 hub genes. The circle node represents lncRNA, the square node represents mRNA, and the triangle node represents TF gene ( p ≤ 0.05). [file peerj-11-15332-s002.pdf]

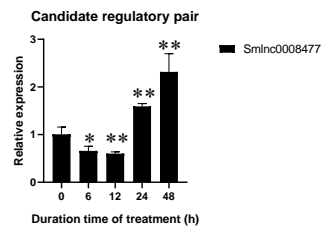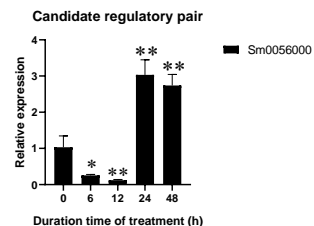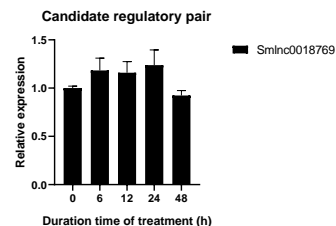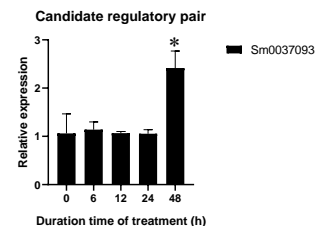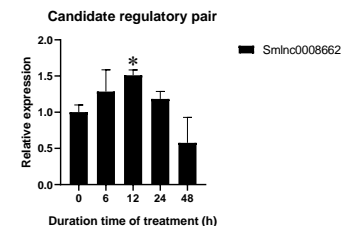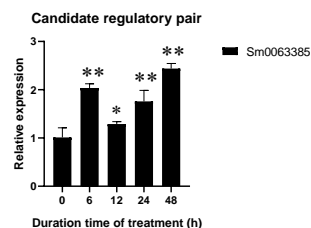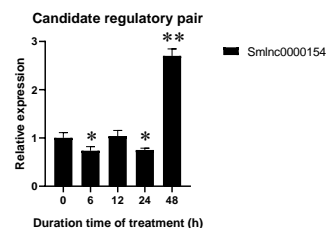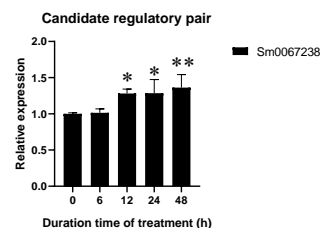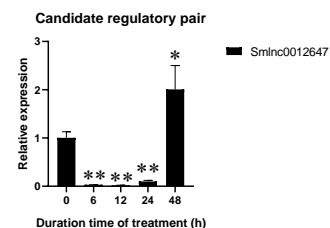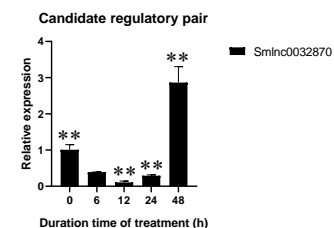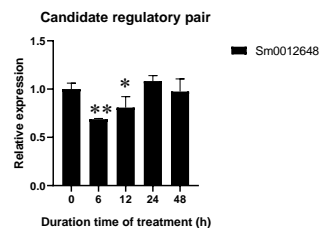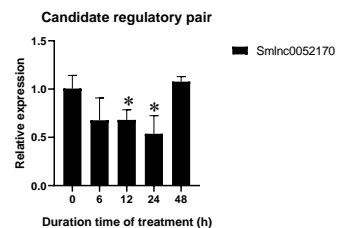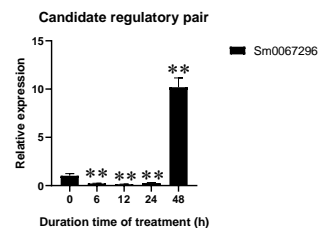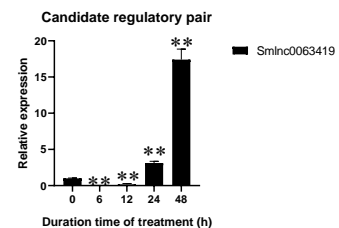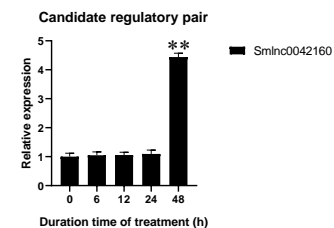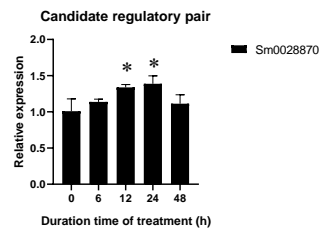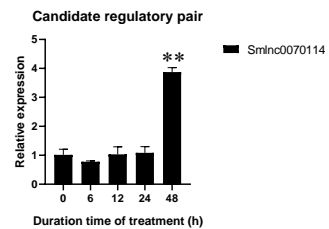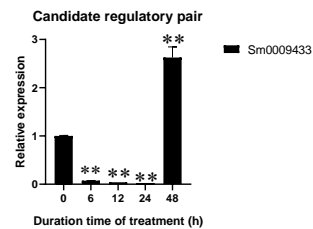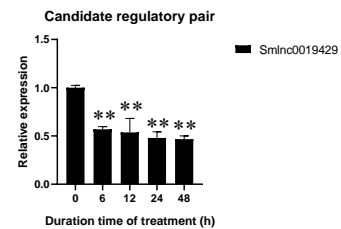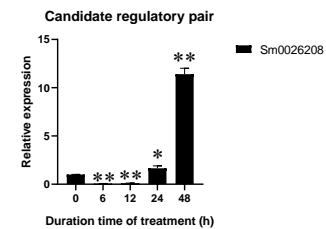

Supplement: Supplemental Information 3 — The expression levels of 11 lncRNAs, 6 mRNAs, and 3 TFs of S. miltiorrhiza treated with MeJA at 6, 12, 24, and 48 h were shown. The expression level of non-treated (0 h) roots of S. miltiorrhiza was arbitrarily set to 1 and the levels induced by MeJA were given relative to this. Fold changes of lncRNA levels were shown. Error bars represent the standard deviation. p value was determined by an unpaired t-test using SPSS (Version 23.0, IBM, USA), p < 0.05 ( ∗) and p < 0.01 ( ∗ ∗) were considered statistically significant. [file peerj-11-15332-s003.pdf]

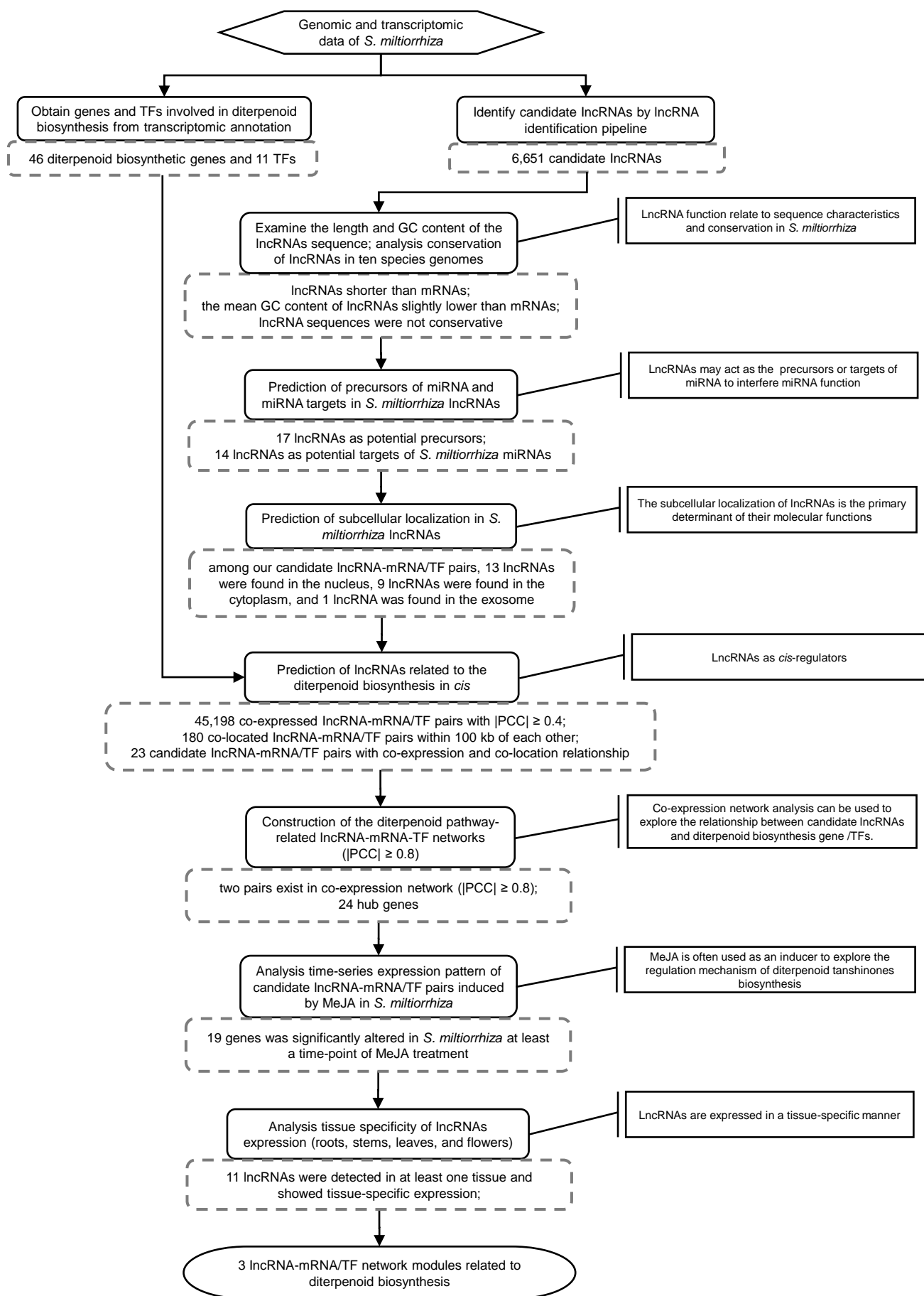

Supplement: Supplemental Information 4 — The figure shows the process of lncRNA function analysis in this study. [file peerj-11-15332-s004.pdf]
